# 

**Published:** 1889-06-29

**Authors:** 


SATURDAY, JUNE 29th, 1889.

**Sir Andrew Clark Taken to Task** - 191

**The Suppression of Vivisection** - 192

**Hydrophobia** - 193

**Pauper's Work** - 194

**The Doctor's Pulpit.**—In Nature's School - 195

**Country Holidays for Town Children** - 196

**The Physician as Naturalist** - 197

**Notes and News** - 198

**Annotations.**—Monkeys as "Topers"—How Children are Poisoned—The Value of Life - 201

**Everybody's Page** - 200

**Editor's Letter-Box.**—The Anti-Vaccinators and the Royal Commission—Cows' Milk and Consumption—Metropolitan Association for Befriending Young Servants - 202

**Her Heart's Mistake.** By E. D. CUMMING. - 203

**The Church and the River** - 204

**Among Soldiers' Children** - 205

**Amusements and Relaxation** - 206

**Scraps and Gleanings** - 206

EXTRA SUPPLEMENT—THE NURSING MIRROR.

**En Passant.**—Marylebone Infirmary—Dublin Nursing Institution—The Nightingale School—A Colonial Nurse—The Pension Fund—Short Items—Registration - xlvii
**A First Experience**—Part II. - xlviii
**Presented** - xlviii
**Everybody's Opinion.**—A Plea for Nurses—The Registration of Nurses - xlix
**Appointments.—Meetings.—Vacancies** - l
**Notes and Queries** - l
**A First Operation** - l

